# Investigating AP-2 and YY1 protein expression as a cause of high HER2 gene transcription in breast cancers with discordant HER2 gene amplification

**DOI:** 10.1186/bcr2461

**Published:** 2009-12-21

**Authors:** Desmond G Powe, Gulfareen Akhtar, Hany Onsy Habashy, Tarek Abdel-Fatah, Emad A Rakha, Andrew R Green, Ian O Ellis

**Affiliations:** 1 Department of Cellular Pathology, Queen's Medical Centre, Nottingham University Hospitals NHS Trust, Derby Road, Nottingham NG7 2UH, UK; 2 Department of Pathology, School of Molecular Medical Sciences, Nottingham University Hospitals NHS Trust and University of Nottingham, Derby Road, Nottingham NG7 2UH, UK; 3 Department of Histopathology, Faculty of Medicine, Mansoura University, Mansoura, Egypt

## Abstract

**Introduction:**

Candidacy for anti-HER2 adjuvant therapy in breast cancer is assessed using tumour HER2 status but recently it has been proposed that the transcription factors AP-2α and YY1 may cause Her2 protein overexpression independently of gene amplification.

**Methods:**

We characterised AP-2α/β, AP-2α and YY1 with HER2 gene and protein expression, other relevant biomarkers, and clinical outcome using tissue microarrays (TMAs) and immunohistochemistry in a large (n = 1,176) clinically annotated series of early stage operable breast cancer. The associations and prognostic independence of AP-2 and YY1 was assessed in all patients and an oestrogen receptor negative subgroup.

**Results:**

Nuclear expression of AP-2α/β, AP-2α and YY1 was detected in 23%, 44% and 33% of cases respectively. AP-2α/β significantly correlated with YY1 and both markers were increased in luminal oestrogen receptor (ER) positive tumours of small size and low grade but only AP-2α/β correlated with good prognosis breast cancer specific survival and disease free interval (BCSS and DFI). These characteristics were lost in oestrogen receptor negative patients. AP-2α also correlated with luminal-type tumours but not with YY1 expression or good prognosis. AP-2α and YY1 showed a significant correlation with Her2 protein expression and in addition, YY1 correlated with HER2 gene expression. Discordant HER2 gene and protein expression was identified in six cases (0.71% of the study group) with four of these showing AP-2α but absence of AP-2α/β and YY1 expression.

**Conclusions:**

AP-2α/β and YY1 are markers of good prognosis principally due to their association with oestrogen receptor but are not independent predictors. Discordant HER2 protein/gene expression is a rare event that is not always explained by the actions of AP-2 and YY1.

## Introduction

HER2 amplification occurs in approximately 20% breast cancer patients and is generally associated with poor prognosis compared to HER2 negative counterparts [1,2]. Over-expression of HER2 is generally seen in high grade breast cancers and is associated with aggressive tumour growth characteristics including increased proliferation [3], enhanced tumorigenicity [4] and metastatic potential [5]. These adverse properties can be ameliorated by blockade of HER2 protein with the humanised monoclonal antibody, trastuzumab, and is targeted at patients that show HER2 amplification [6]. Furthermore, it has been recently shown that HER2 positive node negative patients with low grade breast cancer also benefit from trastuzumab therapy [7] but its increased use has to be balanced by potential toxicity [8].

Over-expression of HER2 protein is usually accounted for by gene amplification. Detection of HER2 protein is routinely performed using immunohistochemistry (IHC) with visual assessment where a score of 3+ assumes HER2 oncogene amplification [9]. In equivocal cases scoring 2+ gene status is assessed using the gold standard fluorescent *in situ *hybridisation or chromogenic *in situ *hybridisation techniques [10]. Recent evidence has suggested that increased transcription of HER2 protein can occur due to activation of the *HER*2 promoter by transcriptional factors, including activating protein-2 (AP-2) and Yin Yang protein-1 (YY1) [11]. The AP-2 family contains five homologous 50 kDa proteins encoded by individual genes, which regulate transcription through interaction with several nuclear factors associated with specific genetic programs [12]. AP-2 proteins have multi-functional roles, including control of normal cell growth and differentiation, and also carcinogenesis including breast cancer [13]. At least four binding sites have been identified for AP-2 in the promoter of the HER2 gene [13,14] and some studies have shown a correlation between the expression of AP-2 with HER2 over-expression in breast cancer cell lines [12-15] suggesting that AP-2 and its neutralisation could be a means of down regulating HER2 [15].

The Yin Yang protein (YY1) is a conserved zinc-finger DNA-binding phosphoprotein transcriptional factor that regulates initiation, activation and repression of transcription for a number of genes connected with cell growth, development and differentiation [16-18]. YY1 has been reported to regulate the oncogene retinoblastoma protein Rb [19] and CBP [20] and furthermore, binds to the ubiquitous alpha component present in replication-dependent histones. Because of its role in chromatin remodelling, involving interaction with histone-deacetylase (HDAC) [21], YY1 may be associated with global gene expression and has received interest as a potential therapeutic target in human cancer [22-24]. Moreover, YY1 has recently been identified as a new cofactor stimulating AP-2 transcriptional activity [25].

In the current study, we used a large (n = 1,176) and well characterised series of patients with early stage invasive breast cancer to investigate the frequency of discordant HER2 gene and protein levels and to evaluate the role of AP-2α/β, AP-2α and YY1 in these tumours. The implication of amplification-independent HER2 overexpression is that patients with equivocal Her2 protein expression (Her2 score 2+) might be denied clinical anti-HER2 adjuvant therapy (AT). In addition, the clinicopathological value of AP-2 and YY1 in breast cancer was assessed in view of the previous conflicting reports and its proposed role as a possible therapeutic target in breast cancer.

## Materials and methods

This study was approved by the Nottingham Research Ethics Committee 2 under the title 'Development of a molecular genetic classification of breast cancer'. Production of the study and analysis of the data was independent of the funding bodies associated with this project.

### Patient selection

Tissue microarrays (TMAs) containing 1,176 primary operable breast carcinoma cases derived from the Nottingham Tenovus Primary Breast Carcinoma Series were used as previously reported [26]. The cohort comprised women aged 70 years or less, who presented between 1986 and 1999. This well-characterized resource contains information on patients' clinical and pathological data including histologic tumour type, primary tumour size, lymph node status, histologic grade, and data on other breast cancer relevant biomarkers. Patients within the good prognostic group (Nottingham Prognostic Index (NPI) ≤ 3.4) did not receive adjuvant therapy (AT). Hormonal therapy (HT) was prescribed to patients with ER-α+ tumours and NPI scores of >3.4 (moderate and poor prognostic groups). Pre-menopausal patients within the moderate and poor prognostic groups were candidates for CMF (Cyclophosphamide, Methotrexate, and 5-Flourouracil) chemotherapy. Conversely, postmenopausal patients with moderate or poor NPI and ER-α+ were offered HT, while ER-α-patients received CMF if fit. Survival data including survival time, disease-free interval (DFI) and development of loco-regional and distant metastases (DM) were maintained on a prospective basis. Median follow up was 124 months (range 1 to 233). Breast cancer specific survival (BCSS) was defined as the time (in months) from the date of the primary surgical treatment to the time of death from breast cancer. DFI was defined as the interval (in months) from the date of the primary surgical treatment to the first loco-regional or distant metastasis.

### Immunohistochemistry

Two antibodies were selected for detection of AP-2; a mouse monoclonal antibody was used to recognise the AP-2α/β isoforms (Abcam, Cambs, UK; clone A6/2/2; dilution 1:700), and a polyclonal rabbit antibody recognising only the AP-2α isoform (Active Motif, Carlsbad, USA; dilution 1:100) was also used. Both AP-2 antibodies and the anti-YY1 antibody (Santa Cruz Biotechnology, Santa Cruz, CA, USA; clone H-10; dilution 1:100) were optimised for immunohistochemistry (IHC) on full face excisional breast tissue sections to assess homogeneity of staining. Subsequently, a 13-slide series (n = 1,176 cases) comprising 4 μm thick formalin fixed paraffin embedded breast cancer tissue TMAs were immunostained using microwave antigen retrieval in 0.01 M pH 6.0 citrate buffer (AP2 antibodies) or 0.01 M pH 8.0 EDTA (YY1). An indirect labelled streptavidin avidin biotin technique (LSAB) technique with diaminobenzidine (DAB) chromogen was performed using a DakoCytomation Techmate 500 Plus (DakoCytomation, Cambridge, UK) automatic immunostainer as previously described [27]. Negative controls were performed by omitting the primary antibody and substitution with diluent. Subsequently, immunostained sections were counterstained in haematoxylin. After immunohistochemistry, cases were assessed using the H-score (histochemical score) to measure staining intensity and the percentage of stained tumour cells corresponding to each intensity [28]. The cases were scored without knowledge of the patient outcome by three of the authors (DGP, GA, HOH) and the resulting data were categorised using thresholds set by reference to a frequency distribution histogram plotted for AP-2 and YY1 proteins and by to the median H-scores. The median H-score of 100 or greater was categorised as positive for AP-2 and YY1 staining. In addition, AP-2 and YY1 were characterised in breast cancer by investigating their association with other breast tumour relevant markers using cut-off thresholds as previously described [26,29].

Her2 scoring was performed on replicate 4 μm thick breast cancer TMAs using a Ventana BenchMark immunostainer (Roche Diagnostics, Surrey, UK) with the Pathway HER2/neu (4B5) rabbit monoclonal antibody according to the manufacturer's instructions. Breast carcinomas were microscopically assessed to determine the level of Her2 protein expression using the manufacturers threshold criteria based on the recommendations of the American Society of Clinical Oncologists and the College of American Pathologists [2]. Intensity and pattern of membrane staining seen in at least 10% of cells was scored: 1+ = non-amplified, 3+ = gene amplification, and 2+ = equivocal result and warranted further investigation of amplification status using an INFORM HER2/neu DNA probe with a silver *in situ *hybridisation (SISH) technique according to the manufacturers instructions (Roche Diagnostics). Sections were counterstained in haematoxylin and all reagents were supplied by Roche Diagnostics. The sections were initially screened using a Nikon E600 microscope (Nikon, Surrey, UK) fitted with an ×20 and ×40 objective lens. The number of silver signals corresponding to the gene copy number of HER2 and chromosome 17 (Chr17) was counted in malignant breast epithelial cells only using a quantitative method. For each tumour, a HER2/Chr17 ratio was determined from the average number of signals located in at least 20 cells from replicate sections. Cases that failed to show signal in normal cells adjacent to neoplastic tissue were excluded. Cases with a HER2/Chr17 ratio less than1.8 were deemed non-amplified whereas ratio's greater than 2.2 were designated as amplified.

### Statistical analysis

Statistical analysis was performed using SPSS 15.0 statistical software (SPSS Inc, Chicago, IL, USA). Association between AP-2 and YY1 protein expression with different clinicopathological parameters was evaluated in the entire patient series and in an ER negative patient cohort only using the Pearson Chi-square or Fisher's exact test. Standard cut-off values for the different biomarkers, needed to determine categorical scores before statistical analysis, were the same as those published in previous studies [26,27,30]. Cumulative survival probabilities were estimated using the Kaplan-Meier method with a log rank test to assess significance between different survival rates. Patients that died due to causes other than breast cancer were censored during survival analysis. Multivariate analysis for survival was performed using the Cox proportional hazard regression model. Each variable was assessed in univariate analysis as a continuous and categorical variable and the two models were compared using an appropriate likelihood ratio test. Hazard ratios (HR) and 95% confidence intervals (95% CI) were estimated for each variable. All tests were two-sided with a 95% CI and significant *P *value was <0.05.

## Results

### Characterisation of AP-2 and YY1 positive tumours and their association with tumour-relevant biomarkers in the entire patient series

**Figure 1:**
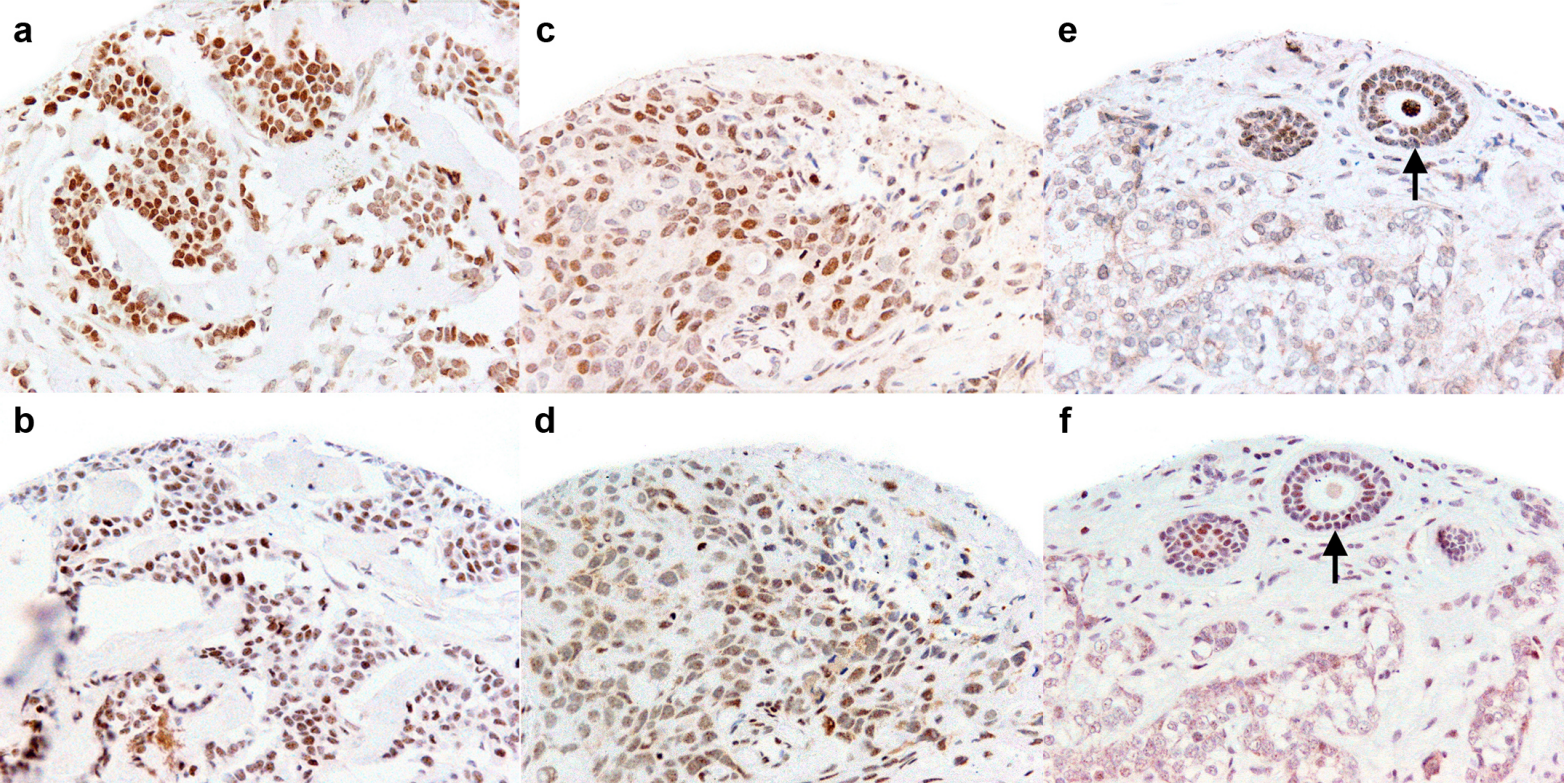
**AP-2 α/β (a, c, e) and YY1 (b, d, f) immunostaining**. Tumour nuclear staining varied from homogeneously strong (a, b) to a mixed (c, d) pattern but was intense in normal glandular tissue (e, f) (arrow) (×20 magnification).

AP-2 and YY1 immunostaining showed predominant nuclear localisation with variable cytoplasmic staining (Figure 1).

**AP-2α/β isoforms:** Informative data were obtained on 1,176 tissue cores of which 22.8% showed AP-2 expression. AP-2α/β and YY1 expression was positively correlated (χ = 167.5, *P *< 0.001).

High AP-2α/β nuclear expression was significantly increased in small (≤ 1.5 cm) compared to large tumours (*P *< 0.001) of low tumour grade (*P *< 0.001). Positive AP-2α/β expression was more frequent in lobular breast cancer (BC) compared to negative cases (17.1% compared to 8.3%, respectively) showing good NPI (*P *< 0.001) and reduced vascular invasion (*P *= 0.036). Patients' characteristics are summarized in Table 1.

**Table 1:** Patients' characteristics showing AP-2 protein expression

	AP-2 (α and β isoforms)			AP-2 (α isoform)		
**Variable**	**Positive (%)**	**Negative (%)**	**χ 2** **(*P*-value)**	**Positive (%)**	**Negative (%)**	**χ 2** **(*P*-value)**
**Patients age**						
**<40**	16 (6.0)	69 (7.6)	2.381 (0.497)	45 (8.7)	35 (8.3)	0.225 (0.973)
**40-50**	69 (25.7)	244 (26.9)		160 (30.8)	134 (31.7)	
**51-60**	79 (29.5)	285 (31.4)		169 (32.6)	133 (31.4)	
**>60**	104 (38.8)	310 (34.1)		145 (27.9)	121 (28.6)	
**Primary tumour size**						
**≤ 1.5 cm**	112 (41.6)	274 (30.3)	12.125 (<0.001)	252 (48.4)	215 (51.6)	0.943 (0.332)
**>1.5 cm**	157 (58.4)	631 (69.7)		269 (51.6)	202 (48.4)	
**Lymph node Stage**						
**1**	177 (65.8)	554 (61.3)	5.382 (0.068)	331 (63.5)	249 (59.6)	1.949 (0.377)
**2**	78 (29.0)	263 (29.1)		143 (27.4)	132 (31.6)	
**3**	14 (5.2)	87 (9.6)		47 (9.0)	37 (8.8)	
**Grade**						
**1**	60 (22.3)	137 (15.2)	43.672 (<0.001)	66 (12.7)	73 (17.5)	4.476 (0.107)
**2**	124 (46.1)	274 (30.3)		160 (30.7)	126 (30.2)	
**3**	85 (31.6)	493 (54.5)		295 (56.6)	218 (52.3)	
**NPI**						
**Poor**	25 (9.3)	224 (24.8)	35.333 (<0.001)	85 (16.3)	84 (20.1)	3.574 (0.167)
**Moderate**	130 (48.3)	517 (57.1)		303 (58.2)	219 (52.4)	
**Good**	114 (42.4)	164 (18.1)		133 (25.5)	115 (27.5)	
**Development of Distant Metastasis**						
**No**	193 (73.1)	611 (68.0)	2.528 (0.112)	341 (66.1)	302 (71.7)	3.436 (0.064)
**Positive**	71 (26.9)	288 (32.0)		175 (33.9)	119 (28.3)	
**Development of Recurrence**						
**No**	165 (62.5)	504 (56.4)	3.069 (0.08)	294 (57.8)	248 (60.6)	1.969 (0.374)
**Positive**	99 (37.5)	389 (43.6)		215 (42.2)	161 (39.4)	
**Vascular invasion**						
**No**	168 (63.2)	495 (55.0)	6.674 (0.036)	286 (55.5)	220 (52.4)	0.949 (0.622)
**Probable**	21 (7.9)	109 (12.1)		61 (11.8)	52 (12.4)	
**Definite**	77 (28.9)	296 (32.9)		168 (32.6)	148 (35.2)	
**Tumour type**						
**Ductal/NST**	129 (49.0)	551 (61.5)	29.22 (<0.001)	326 (63.9)	252 (60.3)	15.31 (0.032)
**Lobular**	45 (17.1)	74 (8.3)		46 (9.0)	35 (8.4)	
**Tubular and Tubular mixed**	61 (23.2)	178 (19.9)		81 (15.9)	90 (21.5)	
**Medullary**	4 (1.5)	29 (3.2)		22 (4.3)	7 (1.7)	
Other special types*	8 (3.0)	12 (1.3)		7 (1.4)	7 (1.7)	
Mixed**	16 (6.1)	47 (5.2)		26 (5.1)	24 (5.7)	
**Menopausal status**						
**Premenopausal**	103 (38.4)	355 (39.1)	0.038 (0.845)	212 (40.8)	171 (40.4)	0.017 (0.896)
**Postmenopausal**	165 (61.6)	553 (60.9)		307 (59.2)	252 (59.6)	

Two different AP-2 antibodies were used; one recognised AP-2α and β isoforms while the other recognised only the AP-2α isoform. *Includes Mucoid, invasive cribriform and invasive papillary carcinoma. **Includes ductal/No special type (NST) mixed with lobular or special types. NPI = Nottingham Prognostic Index.

AP-2α/β nuclear staining positively correlated with the hormonal receptors [oestrogen receptor-α (ER) (*P *< 0.001), progesterone receptor (PgR) (*P *< 0.001), the luminal-associated marker CK19 (*P *= 0.007), and with Bcl2 (*P *= 0.042). Significant inverse correlations were observed between AP-2 and HER4 (*P *= 0.001), Mib1 (*P *= 0.005), EGFR (*P *= 0.045), and the basal markers CK5/6 (*P *= 0.006) and CK14 (*P *= 0.011). Details of the associations with other biomarkers are shown in Table 2.

**Table 2:** Association of two AP-2 isoforms with cancer relevant biomarkers

	AP-2 (α and β isoforms)				AP-2 (α isoform)			
**Biomarker**	**Negative** **(%)**	**Positive** **(%)**	**χ^2^**	***P*-value**	**Negative**	**Positive**	**χ^2^**	***P*-value**
**ER**								
**Negative**	293 (34.6)	45 (18.5)	22.70	<0.001	248 (34.4)	102 (26.0)	5.287	0.02
**Positive**	555 (65.4)	198 (81.5)			472 (65.6)	291 (74.0)		
**PgR**								
**Negative**	410 (48.3)	72 (30.0)	25.524	<0.001	358 (49.9)	173 (44.0)	0.514	0.473
**Positive**	438 (51.7)	168 (70.0)			359 (50.1)	220 (56.0)		
**CK19**								
**Negative**	87 (10.0)	11 (4.5)	7.349	0.007	63 (12.6)	31 (7.7)	5.811	0.016
**Positive**	785 (90.0)	236 (95.5)			436 (87.4)	372 (92.3)		
**HER3**								
**Negative**	77 (10.7)	24 (11.9)	0.222	0.638	40 (9.4)	20 (5.7)	3.730	0.053
**Positive**	642 (89.3)	178 (88.1)			387 (90.6)	333 (94.3)		
**HER4**								
**Negative**	131 (18.3)	60 (29.1)	11.491	0.001	82 (19.0)	39 (11.3)	8.776	0.003
**Positive**	586 (81.7)	146 (70.9)			349 (81.0)	307 (88.7)		
**E-cadherin**								
**Negative**	345 (40.5)	85 (34.6)	2.871	0.09	179 (36.3)	142 (35.7)	0.038	0.846
**Positive**	506 (59.5)	161 (65.4)			314 (63.7)	256 (64.3)		
**EGFR**								
**Negative**	582 (78.9)	182 (85.0)	4.004	0.045	361 (80.6)	286 (78.4)	0.612	0.434
**Positive**	156 (21.1)	32 (15)			87 (19.4)	79 (21.6)		
**CK5/6**								
**Negative**	713 (81.5)	224 (88.9)	7.65	0.006	399 (79.3)	346 (86.3)	7.457	0.006
**Positive**	162 (18.5)	28 (11.1)			104 (20.7)	55 (13.7)		
**CK14**								
**Negative**	736 (85.5)	233 (94.0)	6.456	0.011	426 (86.8)	361 (90.9)	3.787	0.052
**Positive**	125 (14.5)	15 (6.0)			65 (13.2)	36 (9.1)		
**P53**								
**Negative**	620 (72.6)	189 (77.8)	2.62	<0.106	328 (66.8)	294 (74.1)	5.504	0.019
**Positive**	234 (27.4)	54 (22.2)			163 (33.2)	103 (25.9)		
**Mib1**								
**Negative**	189 (43.0)	76 (56.7)	7.827	0.005	95 (34.2)	100 (50.5)	12.755	<0.001
**Positive**	251 (57.0)	58 (43.3)			183 (65.8)	98 (49.5)		
**Bcl2**								
**Negative**	43 (12.8)	7 (6.0)	4.139	0.042	18 (9.8)	24 (13.2)	1.044	0.307
**Positive**	292 (87.2)	110 (94.0)			166 (90.2)	158 (86.8)		

ER: oestrogen receptor; PgR: Progesterone receptor; CK-: Cytokeratin; EGFR: epidermal growth factor receptor.

**AP-2α isoform:** An antibody recognising AP-2α isoform was selected to allow comparison with a previous study reported by Allouche et al [11]. The pattern of staining using the AP-2α isoform-specific antibody was similar to that seen using the AP-2α/β antibody but no significant association was seen with YY1 (χ = 3.297, *P *< 0.069). Informative data were obtained on up to 942 cases with 44% showing positive staining. AP-2α expression showed no significant association with patient age, tumour size, grade, lymph node stage, prognosis using NPI, formation of distant metastases, tumour recurrence or vascular invasion. The frequency of positive AP-2α expression was two-fold more prevalent in medullary BC compared to negative cases. Patients' characteristics are summarized in Table 1.

AP-2α nuclear staining positively correlated with the hormonal receptor ER (*P *= 0.02), and the luminal-associated marker CK19 (*P *= 0.016). Significant inverse correlations were observed between AP-2α and HER4 (*P *= 0.003), Mib1 (*P *< 0.001), and the basal marker CK5/6 (*P *= 0.006). Details of the association between with other biomarkers are shown in Table 2.

**YY1:** Informative data were obtained on 1,159 tissue cores with 33.3% being positively categorized for YY1 expression. High YY1 nuclear expression was significantly increased in small (≤ 1.5 cm) (*P *= 0.028) low grade (*P *< 0.001) tumours with good NPI (*P *< 0.001). Positive YY1 expression was more frequent in lobular and tubular BC compared to negative cases. No significant association was found with respect to patient age, lymph node stage, formation of distant metastases, tumour recurrence or vascular invasion. Patients' characteristics are summarized in Table 3.

**Table 3:** Patients' characteristics showing YY1 protein expression

	YY1		
**Variable**	**Positive (%)**	**Negative (%)**	**χ^2^** **(*P*-value)**
**Patients age**			
	26 (6.7)	53 (6.9)	1.454 (0.693)
	110 (28.5)	196 (25.4)	
	119 (30.8)	242 (31.3)	
	131 (33.9)	282 (36.5)	
**Primary tumour size**			
**≤ 1.5 cm**	144 (37.6)	241 (31.1)	4.817 (0.028)
**>1.5 cm**	239 (62.4)	533 (68.9)	
**Lymph node Stage**			
**1**	234 (61.1)	492 (63.6)	1.982 (0.371)
**2**	118 (30.8)	209 (27.0)	
**3**	31 (8.1)	72 (9.3)	
**Grade**			
**1**	85 (22.2)	113 (14.6)	46.984 (<0.001)
**2**	162 (42.3)	220 (28.5)	
**3**	136 (35.5)	440 (56.9)	
**NPI**			
**Poor**	49 (12.8)	193 (24.9)	17.284 (<0.001)
**Moderate**	195 (50.9)	444 (57.4)	
**Good**	139 (36.3)	137 (17.7)	
**Development of Distant Metastasis**			
**No**	274 (71.5)	521 (68.2)	1.343 (0.246)
**Positive**	109 (28.5)	243 (31.8)	
**Development of Recurrence**			
**No**	226 (59.2)	435 (57.4)	0.328 (0.567)
**Positive**	156 (40.8)	323 (42.6)	
**Vascular invasion**			
**No**	232 (60.7)	417 (54.4)	4.45 (0.108)
**Probable**	37 (9.7)	94 (12.3)	
**Definite**	113 (29.6)	256 (33.4)	
**Tumour type**			
**Ductal/NST**	195 (51.2)	474 (62.3)	29.509 (<0.001)
**Lobular**	53 (13.9)	59 (7.8)	
**Tubular and Tubular mixed**	99 (26.0)	140 (18.4)	
**Medullary**	5 (1.3)	29 (3.8)	
Other special types*	8 (2.1)	11 (1.4)	
Mixed**	19 (5)	45 (5.9)	
**Menopausal status**			
**Premenopausal**	160 (41.5)	284 (36.7)	2.417 (0.12)
**Postmenopausal**	226 (58.5)	489 (63.3)	

*Includes Mucoid, invasive cribriform and invasive papillary carcinoma. **Includes ductal/No special type (NST) mixed with lobular or special types. NPI = Nottingham Prognostic Index.

Positive YY1 nuclear staining was associated with ER (*P *< 0.001), PgR (*P *< 0.001), CK19 (*P *= 0.037), and Bcl2 (*P *< 0.001). Significant inverse correlations were found between YY1 and HER4 (*P *< 0.001), CK5/6 (*P *= 0.015), CK14 (*P *= 0.023), p53 (*P *= 0.002), and MIB1 (*P *= 0.035). Details of the associations with other biomarkers are shown in Table 4.

**Table 4:** The association of YY1 protein levels were compared with cancer relevant biomarkers in breast tumours

	YY1			
**Biomarker**	**Negative**	**Positive**	**χ^2^**	***P*-value**
**ER**				
**Negative**	248 (34.4)	64 (17.9)	31.912	<0.001
**Positive**	472 (65.6)	294 (82.1)		
**PgR**				
**Negative**	358 (49.9)	120 (33.7)	25.343	<0.001
**Positive**	359 (50.1)	236 (66.3)		
**CK19**				
**Negative**	77 (10.4)	24 (6.6)	4.370	0.037
**Positive**	661 (89.6)	341 (93.4)		
**HER3**				
**Negative**	68 (11.1)	37 (12.5)	0.388	0.534
**Positive**	543 (88.9)	258 (87.5)		
**HER4**				
**Negative**	104 (16.9)	85 (28.7)	16.938	<0.001
**Positive**	511 (83.1)	211 (71.3)		
**E-cadherin**				
**Negative**	294 (40.8)	127 (35.2)	3.170	0.075
**Positive**	427 (59.2)	234 (64.8)		
**EGFR**				
**Negative**	503 (79.7)	251 (81.0)	0.205	0.651
**Positive**	128 (20.3)	59 (19.0)		
**CK5/6**				
**Negative**	599 (80.6)	315 (86.5)	5.947	0.015
**Positive**	144 (19.4)	49 (13.5)		
**CK14**				
**Negative**	623 (85.3)	325 (90.3)	5.183	0.023
**Positive**	107 (14.7)	35 (9.7)		
**P53**				
**Negative**	508 (70.2)	281 (78.9)	9.318	0.002
**Positive**	216 (29.8)	75 (21.1)		
**Mib1**				
**Negative**	168 (41.7)	91 (51.1)	4.450	0.035
**Positive**	235 (58.3)	87 (48.9)		
**Bcl2**				
**Negative**	230 (38.9)	72 (24.3)	18.556	<0.001
**Positive**	362 (61.1)	224 (75.7)		

ER = oestrogen receptor; PgR = Progesterone receptor; CK- = Cytokeratin; EGFR = epidermal growth factor receptor.

### Characterisation of AP-2 and YY1 positive tumours and their association with tumour-relevant biomarkers in ER-negative patients

Due to the significant correlation found between AP2 with ER and the predicted good clinical outcome in hormonal-positive patients, these associations were investigated in ER-negative cases to test the dependency on ER status.

**AP-2α/β isoforms:** AP-2α/β protein expression was studied in 309 ER negative patients and showed no significant association with either tumour size, lymph node stage, histological grade, development of metastasis, vascular invasion, tumour type, menopausal status, PgR, CK19, HER3, HER4, E-cadherin, EGFR, CK5/6, CK14, p53 or Bcl2 (data not shown). However, AP-2α/β protein expression showed significant inverse associations with the proliferation marker Mib1 (χ^2 ^= 4.671, *P *= 0.031) and good prognosis measured using NPI (χ^2 ^= 6.428, *P *= 0.04).

**AP-2α isoform:** AP-2α/β protein expression was studied in 266 ER negative patients and showed no significant association with tumour size, lymph node stage, histological grade, prognosis (NPI), development of metastasis, vascular invasion, tumour type, menopausal status, PgR, HER3, E-cadherin, EGFR, CK14, p53 or Bcl2 (data not shown). However, AP-2α protein expression showed significant positive association with CK19 (χ^2 ^= 4.982, *P *= 0.026) and HER4 (χ^2 ^= 5.885, *P *= 0.015), and a significant negative association with the basal marker CK5/6 (χ^2 ^= 5.569, *P *= 0.018) and the proliferation marker Mib1 (χ^2 ^= 8.521, *P *= 0.004).

### Association of AP-2 and YY1 with clinical outcome

The AP-2 and YY1 antibodies were tested using Kaplan Meier plots to model clinical outcome, and using Cox regression analysis to determine their ability to predict clinical outcome measured against tumour size, grade, and lymph node stage.

**Figure 2:**
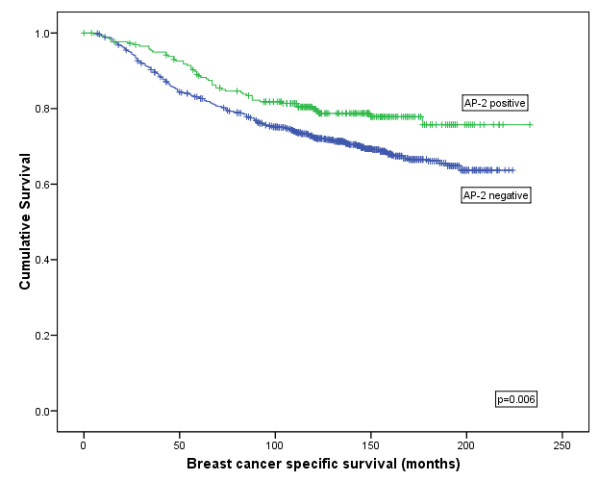
**Kaplan-Meier model comparing breast cancer specific survival in breast cancer patients with AP-2 α/β protein expression**.

**Figure 3:**
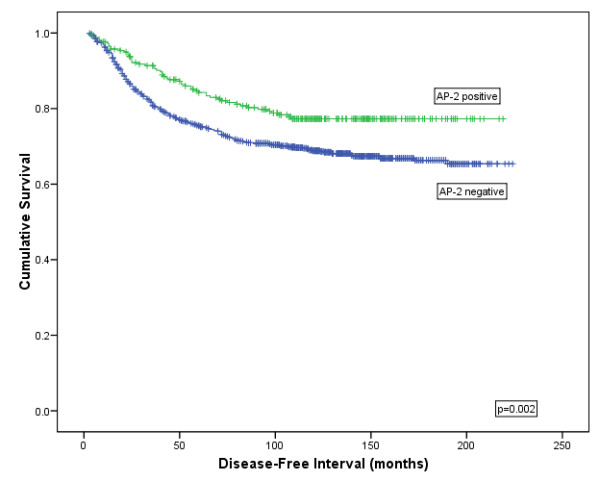
**Kaplan-Meier model comparing disease free interval in breast cancer patients showing AP-2α/β protein expression**.

**AP-2α/β isoforms:** AP-2α/β expression was significantly associated with longer breast cancer specific survival (BCSS) (Log rank (LR) = 7.607, *P *= 0.006; HR = 1.5 (95% CI = 1.1 to 2.01), *P *= 0.006; Figure 2) and an increased disease free interval (DFI) (LR = 9.201, *P *= 0.002; HR = 1.565 (95% CI:1.168 to 2.096), *P *= 0.003; Figure 3) but not for distant metastasis (DM) formation or tumour recurrence. The AP-2α/β specific antibody was not found to be an independent predictor of survival using Cox regression (*P *= 0.397; HR = 0.880 (95% CI: 0.654 to 1.184)).

**AP-2α isoform:** AP-2α showed no significant association with improved BCSS (χ^2 ^= 0.888, *P *= 0.346), DFI (χ^2 ^= 1.557, *P *= 0.212), DM (χ^2 ^= 1.838, *P *= 0.175) or tumour recurrence (χ^2 ^= 0.207, *P *= 0.649) using Kaplan-Meier modelling. The AP-2α specific antibody was not found to be an independent predictor of survival using Cox regression (*P *= 0.866; HR = 0.978 (95% CI: 0.751 to 1.272)).

**Figure 4:**
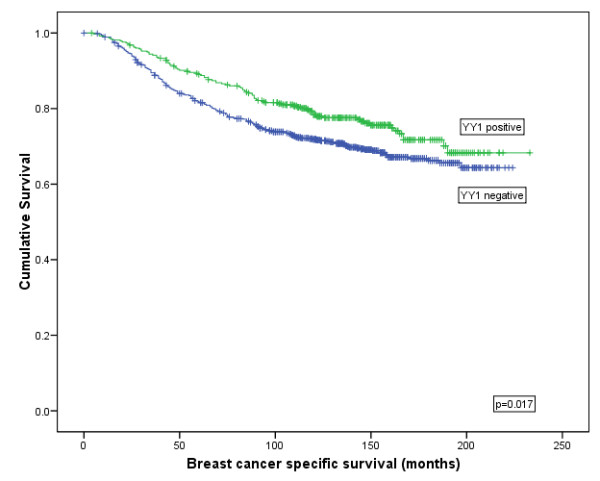
**Kaplan-Meier model comparing breast cancer specific survival in breast cancer patients showing YY1 protein expression**.

**Figure 5:**
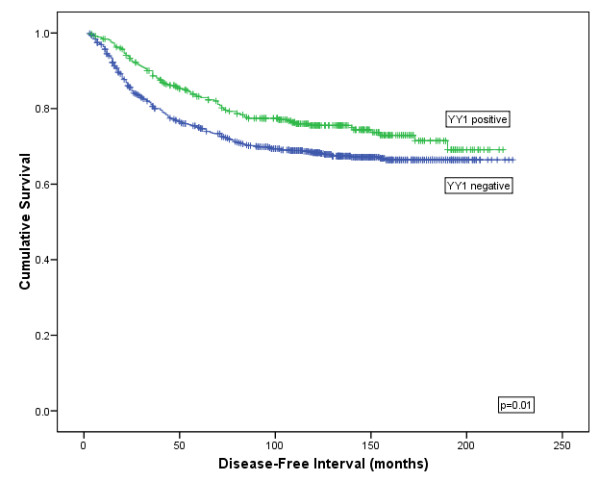
**Kaplan-Meier model comparing disease free interval in breast cancer patients showing YY1 protein expression**.

**YY1:** YY1 showed a significant association with improved BCSS (χ^2 ^= 5.565, *P *= 0.018; Figure 4) and DFI (χ^2 ^= 6.576, *P *= 0.010; Figure 5) but not for DM (χ^2 ^= 2.443, *P *= 0.118) or tumour recurrence (χ^2 ^= 0.438, *P *= 0.508) using Kaplan Meier modelling. YY1 was not found to be an independent predictor of survival using Cox regression (*P *= 0.866; HR = 0.978 (95% CI:0.751 to 1.272)).

### Correlation between HER2 protein and gene expression and the role of AP-2 and YY1

**Figure 6:**
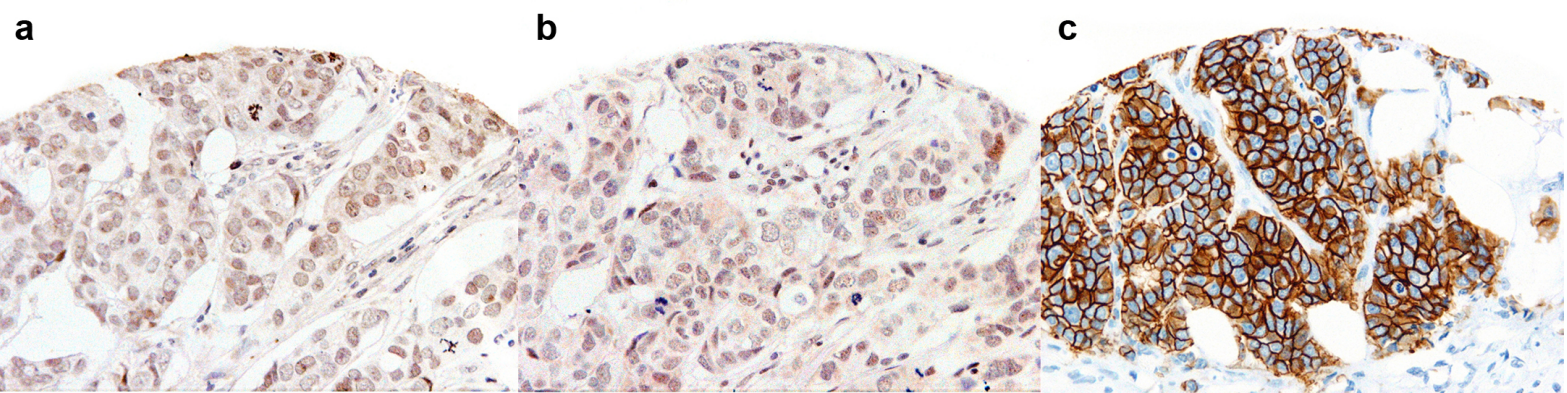
**AP-2α/β, YY1 and Her-2 immunostaining**. Weak AP-2α/β **(a) **and YY1 **(b) **expression but strong (3+) Her-2 staining **(c) **is seen in this tumour. (×20 magnification)

**AP-2α/β isoform:** Her2 protein expression data were available for 679 tumour cores expressing AP-2α/β (Figure 6). AP-2α/β staining was seen in 120/555 (21.6%), 13/34 (38.2%), 5/26 (19.2%), and 13/64 (20.3%) cases scoring 0, 1+, 2+ and 3+ for Her2 membranous staining respectively. Eighteen of 90 (20.0%) patients with 2+ or 3+ Her2 membrane expression were AP-2α/β positive. No significant association was seen between AP-2α/β and Her2 protein staining (Table 5).

**Table 5:** Association between AP-2 protein expression and HER-2 status

		**AP-2 (α and β isoforms)**				**AP-2 (α isoform)**			
		
		**Negative**	**Positive**	**χ^2^**	***P*-value**	**Negative**	**Positive**	**χ^2^**	***P*-value**
**HER-2** **Gene** **Expression**	**Normal**	459 (76.4%)	142 (23.6%)	2.782	0.249	217 (53.6%)	188 (46.4%)	2.664	0.446
	**Amplified**	60 (76.9%)	18 (23.1%)			31 (49.2%)	32 (50.8%)		
	**Gain**	21 (91.3%)	2 (8.7%)			15 (46.9%)	17 (53.1%)		
**HER-2** **Protein** **Expression**	**0**	435 (78.4%)	120 (21.6%)	5.427	0.143	237 (55.4%)	191 (44.6%)	8.550	0.032
	**1+**	21 (61.8%)	13 (38.2%)			62 (66.6)	31 (33.3)		
	**2+**	21 (80.8%)	5 (19.2%)			17 (60.7%)	11 (39.3%)		
	**3+**	51 (79.7%)	13 (20.3%)			37 (45.1%)	45 (54.9%)		

Two different AP-2 antibodies were used; one recognised AP-2α and β isoforms while the other recognised only the AP-2α isoform. Significance level *P *= 0.05.

HER2 gene amplification showed a highly significant positive association with Her2 (3+) protein scoring (*P *< 0.001) and was detected in 78/702 (11.1%). AP-2α/β staining was present in 18/78 (23.1%) gene amplified cases but showed no significant difference compared to the non-amplified tumours (*P *= 0.249). Details of the association between AP-2α/β and HER2 expression are contained in Table 5.

**AP-2α isoform:** Her2 protein expression data were available for 631 tumour cores expressing AP-2α and showed a significant positive association with AP-2α staining (P = 0.032). AP-2α staining was seen in 31/93 (33.3%), 191/428 (44.6%), 11/28 (39.3%), and 45/64 (54.8%) cases scoring 0, 1+, 2+ and 3+ for Her2 membranous staining respectively. Fifty-six of 110 (50.9%) patients with 2+ or 3+ Her2 membrane expression were AP-2α positive.

HER2 gene amplification was detected in a total of 63/500 (12.6%) cases with 32 (6.4%) of these expressing AP-2α staining. No significant association was found between AP-2α protein expression and HER2 amplification (P = 0.446). Details of the association between AP-2α/β and HER2 expression are contained in Table 5.

**YY1:** Her2 protein expression data were available for 666 tumour cores expressing YY1. YY1 staining was seen in 178/544 (32.7%), 20/33 (60.6%), 7/28 (25.0%), 10/61 (16.4%) cases scoring 0, 1+, 2+ and 3+ for Her2 membranous staining respectively, and showed a significant inverse correlation with Her2 protein expression (*P *< 0.001). Seventeen of 215 (7.9%) patients with positive YY1 staining showed 2+ or 3+ Her2 membrane expression Table 6.

**Table 6:** Association between YY1 nuclear protein expression with HER-2 gene and protein expression levels

		YY1			
		**Negative**	**Positive**	**χ^2^**	***P*-value**
**HER-2 Gene Expression**	**Normal**	315 (40.6%)	190 (49.4%)	13.115	0.004
	**Amplified**	68 (8.8%)	16 (4.2%)		
	**Gain**	13 (1.7%)	7 (1.8%)		
**HER-2 Protein Expression**	**0**	366 (67.3%)	178 (32.7%)	19.882	<0.001
	**1+**	13 (39.4%)	20 (60.6%)		
	**2+**	21 (4.7%)	7 (3.3%)		
	**3+**	51 (11.3%)	10 (4.7%)		

Significance level *P *= 0.05.

HER2 gene amplification was detected in 84/609 (13.8%) cases and showed an inverse correlation with YY1 protein expression (*P *= 0.004). 16/84 (19%) YY1-positive cases also showed HER2 gene amplification. Details of the association between AP-2 and YY1 with HER2 expression are contained in Table 6.

### Cases showing discordant HER2 protein and gene expression and their association with AP-2 and YY1 expression

HER2 SISH gene data were available for 846 cases with 115/842 (13.7%) showing amplification and 32/842 (3.8%) cases showing gene gain. Over-expression (3+) of Her2 protein was seen in 118 cases but only 2 cases (0.24% total cohort), equivalent to 2/118 (1.7%) cases, showed an absence of HER2 gene amplification was seen in just showing (3+) Her2 over-expression. Both cases were AP-2α and AP-2α/β positive but YY1 negative.

A further 5/842 (0.6%) cases showed (3+) Her2 protein over-expression in the presence of HER2 gene gain. Two of these cases were AP-2α positive but AP-2α/β and YY1 negative, one case was AP-2α and AP-2α/β positive but YY1 negative, and one case was negative for all three markers.

An additional 4/842 (0.5%) cases showed equivocal (2+) Her2 protein over-expression in the absence of HER2 gene amplification. Two of these cases were AP-2α positive but AP-2α/β and YY1 negative and two cases were negative for all three markers.

In summary, a total of 6/141 (4.3%) cases showed equivocal or over-expressed Her2 protein in the absence of HER2 gene amplification or gain, representing 0.71% of the total patient cohort.

## Discussion

A number of studies have proposed conflicting roles for AP-2 in breast cancer including a tumour suppressor gene (TSG) function [31,32], or more recently and controversially, that of an oncogene [11]. In the largest study of patients with primary operable breast cancer to date, we characterised two isoforms of the transcription factor AP-2, namely AP-2α/β and AP-2α, and its cofactor YY1. We found differences between the AP-2 isoforms that could explain the conflicting roles reported for AP-2. Importantly, we showed a strong association between AP-2α/β expression and the luminal (ER positive) tumour phenotype that endows these patients with favourable clinical outcome characterised by prolonged survival and longer disease free interval. In contrast the association between AP-2α and ER is weaker and patients with this profile do not have such good prognosis, supporting a tumour suppressor role for AP-2β.

In contrast to luminal breast cancers, HER2 tumours are characterised by an aggressive phenotype and if untreated have a poor prognosis. However, targeted treatment with the humanised monoclonal anti-HER2 antibody, trastuzumab, can significantly improve a patient's outcome [6,8,9]. It has recently been proposed that the transcription factor AP-2α and its cofactor, YY1, can induce *HER2 *gene transcription independently of HER2 gene mutation in breast cancer [11]. For this reason we investigated discordant HER2 gene and protein expression and the association between the transcription factors AP-2 and YY1 and their association with HER2 in a large population of well characterised breast cancer patients. Two different AP-2 antibodies were used; one was specific for AP-2α/β isoforms while the other was specific for the AP-2α isoform only. We found 23% and 44% patients showed high AP-2α/β and AP-2α levels, respectively, which is in the general range (17-42%) [11,32] reported by others. Allouche et al used a percentage scoring technique and a threshold of >80% to determine AP-2α and YY1 positivity, but even though we used a different scoring technique (H-score) with the same AP-2α antibody, we showed good agreement with their study in positively categorizing 44% our patients compared to 42% in the study by Allouche et al [11].

Moreover, Allouche et al proposed that AP-2α can induce Her2 protein expression in patients with absence of HER2 gene amplification because of an observed inverse relationship between breast cancers expressing AP-2α protein and a positive association with HER2 gene amplification [11]. Our findings support Allouche in showing a positive correlation between AP-2α and Her-2 membranous expression but we did not find a significant association between AP-2α and HER2 gene amplification, or between AP-2α and YY1. Similarly, no significant association was identified between AP-2α/β expression and HER2 gene and protein expression. Some of these differences appear to be explained by differences in the patient populations analyzed in Allouche's and our study as only 56% of our study cohort scored 2+/3+ by Her2 IHC compared to 83% identified by Allouche et al. Furthermore, Allouche et al observed 15/55 (27.3%) patients with strong (3+) Her3 IHC staining and 2/15 (13.3%) with absence of HER2 amplification but with strong AP-2α/YY1 expression. In our study, we report 16.2% of our patients had (3+) Her2 IHC staining and just 2/82 (2.4%) of these showed absence of gene amplification; both cases expressed AP-2α but not YY1. Our findings suggest that discordant HER2 expression is a rare event in 3+ Her2 cases and while AP-2α may have a role, further studies are needed to validate this and to study the role of YY1. However, discordant HER2 patients face a possible dilemma in their treatment because the moderate to high levels of HER2 protein expression support candidacy for anti-HER2 AT but contradictory (negative) HER2 gene expression suggest AT refusal. Further studies are needed to assess anti-HER2 AT in this patient group.

Although both AP-2α and AP-2α/β specific antibodies detected tumours with a significant association with ER, the association was stronger when using the AP-2α/β marker as indicated by the strong association with the hormonal receptor PgR and luminal marker CK19. A possible conclusion resulting from this observation is that the AP-2β isoform shows a stronger association with ER. YY1 also showed similar features in associating with luminal type cancers and showed a significant association with AP-2α/β. Consequently, along with others [31-33] we found a significant association between AP-2α/β and good prognosis but this relationship was lost in our ER-negative patient subgroup. ER blockade in ER+ breast cancers reduces AP-2α gene expression but at the same time increases AP-2γ expression [34] and in addition, it has been reported that AP-2α/γ can increase the activity of the ERβ promoter resulting in its increased gene expression [35]. Whereas there is good evidence favouring a TSG function for AP-2α, it has been proposed that AP-2γ is an oncogene affecting multiple growth signalling pathways [36] and functions as an independent predictor of poor survival in patients with breast cancer[37].

In the current study, although the association between nuclear AP-2 expression and good clinical outcome might be explained by its association with ER, there is evidence suggesting that AP-2 influences important biological pathways. In one study, patients with AP-2α positive tumours showed prolonged survival and an increased disease free interval possibly explained by the activity of AP-2α in inducing growth arrest by activation of the cell cycle inhibitor p21WAF1 [38]. Our findings are supportive in showing decreased cell proliferation using the marker Mib1. *In vivo *colonic cell studies suggest AP-2 up-regulates the over-expression of the cell adhesion molecule E-cadherin needed for cell cohesion, while simultaneously decreasing transcription of the matrix metalloproteinase (MMP)-9 [39] involved in migration of tumour cells through adjacent stromal tissue. Our findings and those of others [40] support this in showing increased frequency of AP-2 expression in lobular type breast cancers, generally regarded in having good prognosis. However, our data showed that the AP-2α/β marker was not found to have prognostic independence.

Prior to targeting AP-2 for therapeutic benefit further work is needed to better characterise the function of the AP-2 gene family because of conflicting data concerning its association with tumour development. For instance, the anti-inflammatory drug tolfenamic acid has been shown to inhibit AP-2 and YY1 and in turn this has been shown to down-regulate HER2 mRNA expression [15]. In contrast, our data suggest that the neutralisation of AP-2 and YY1 would have negligible impact on the treatment of HER2 over-expressers and at worst, could actually negate the tumour suppressing benefits of AP-2 as reported by ourselves and others [31,32,35,39].

## Conclusions

Approximately 2% of breast cancer patients express discordant HER2 protein expression independently of HER2 gene amplification. We found that expression of the transcription factors AP-2 and YY1 associated with this in only 0.32% of the entire study group. Further studies are required to determine the response of patients to anti-HER2 therapy when presenting with discordant HER2 gene and protein expression. In support of previous studies, we found that AP-2 and YY-1 correlate with good clinical outcome in patients with breast cancer.

## Abbreviations

AP-2: activator protein-2; AR: androgen receptor; AT: adjuvant therapy; Bcl2: B-cell lymphoma-2; BCSS: breast cancer specific survival; BRCA1: breast cancer gene 1; CBP: CREB binding protein; CI: confidence interval; CK: cytokeratin; DFI: Disease free interval; DM: distant metastasis; EGFR: epidermal growth factor receptor; ER: oestrogen receptor; FOXA1: Forkhead box gene A1; HDAC: histone-deacetylase; IHC: immunohistochemistry; Mib1: antibody that recognises the proliferation antigen Ki67; PgR: progesterone receptor; TMA: tissue microarray; YY1: Yin Yang protein-1.

## Competing interests

The authors declare that they have no competing interests.

## Authors' contributions

DGP conceived and designed the project, participated in the practical work, performed the statistics and drafted the manuscript. GA participated in the practical work, contributed to the manuscript. HOH participated in the practical work, contributed to the manuscript. TA-F, EAR, ARG and IOE contributed to the manuscript. All authors read and approved the final manuscript.
